# The Effects of Tai Chi on Heart Rate Variability in Older Chinese Individuals with Depression

**DOI:** 10.3390/ijerph15122771

**Published:** 2018-12-07

**Authors:** Jing Liu, Huihui Xie, Ming Liu, Zongbao Wang, Liye Zou, Albert S. Yeung, Stanley Sai-chuen Hui, Qing Yang

**Affiliations:** 1 Department of Martial Arts, Shanghai University of Sport, Shanghai 200438, China; xiehuihui1987@163.com (H.X.); cyan810@163.com (Q.Y.); 2 Department of Psychology, School of Kinesiology, University of Michigan, Ann Arbor, MI 48109, USA; 3 Department of Sports, Nanjing University of Science and Technology ZiJin College, Nanjing 210023, China; 4 School of Physical Education, South China University of Technology, Guangzhou 510640, China; liuming@scut.edu.cn; 5 School of Acupuncture and Tuina, University of Chinese Medicine, Anhui 230038, China; wang673099692@hotmail.com; 6 Department of Sports Science and Physical Education, The Chinese University of Hong Kong, Shatin, Hong Kong, China; liyezou123@cuhk.edu.hk (L.Z.); hui2162@cuhk.edu.hk (S.S.-c.H.); 7 Depression Clinical and Research Program, Harvard Medical School, Boston, MA 02114, USA; ayeung@mgh.harvard.edu

**Keywords:** depression, heart rate variability, mind-body, Tai Chi

## Abstract

*Background* Very little research has been done to simultaneously investigate the effects of Tai Chi (TC) on depression and heart rate variability (HRV). This study, therefore, attempted to explore the effects of TC on depression and on HRV parameters. *Methods* Sixty older individuals with depression score of 10 or above (the Geriatric Depression Scale, GDS) were randomly assigned into two groups: TC (*n* = 30) and control group (*n* = 30). Participants in the experimental group participated in a 24-week TC training program (three 60-min sessions per week), whereas individuals in the control group maintained their unaltered lifestyle. Depression and HRV were measured using the GDS and digital electrocardiogram at baseline and after the 24-week intervention. *Results* The TC had produced significant positive chances in depression and some HRV parameters (mean heart rate, RMSSD, HF, LFnorm, and HFnorm) (*p* < 0.05), whereas these positive results were not observed in the control group. *Conclusions* The results of this study indicated that TC may alleviate depression of the elderly through modulating autonomous nervous system or HRV parameters. This study adds to a growing body of research showing that TC may be effective in treating depression of the elderly. Tai Chi as a mild to moderate mind-body exercise is suitable for older individuals who suffer from depression.

## 1. Introduction

Depression is highly prevalent in ageing populations [1] and is projected to be the leading cause of disease burden in both developed and developing countries by 2030 [2]. The World Health Organization estimated 121 million older individuals who are suffering from depression worldwide [3]. Depression symptoms among older individuals are associated with increased risk of death (including suicide), disability, cognitive impairment, and anxiety [4]. Later-life depression is frequently underdiagnosed [5]. reasons for this finding is that elderly patients with depression are reluctant to seek psychological services [6], with the belief that depression or mood symptoms are a normal part of old age [7,8,9].

The research community has recently proposed that heart rate variability (HRV) is a trait maker to objectively assessing mental health (e.g., depression, anxiety, and stress level) [10]. Heart rate variability is a non-invasive approach to measure the specific changes in time-interval between successive heart beats [11]. The accumulated evidence indicates that reduced HRV is possibly associated with a high risk of developing psychological illness like depression [12]. A recently published large-scale cross-sectional study indicated that physical exercise is significantly and meaningfully associated with improved emotional well-being including depression [13]. This encouraging finding suggests that exercise should be considered as part of a healthy lifestyle, particularly for those who suffer from depression [13]. To further confirm the positive results, interventional studies are needed at this current stage.

Tai Chi (TC) is a typical form of mind-body exercise [14], characterized by continuous slow movements [15], deep breathing [16], mental concentration [17], and muscular relaxation [18]. Early studies reported positive effects of TC on depression [18,19]. Furthermore, regular practice in TC has been shown to reduce depression symptoms (behavioral outcome) in aging populations [19,20,21,22,23]. Researchers recently investigated whether changes of HRV parameters can be used as biomarkers to support the positive changes in depression due to mind-body exercises (e.g., Yoga and Tai Chi). As the number of mind-body exercise studies on this topic increased, Zou et al. [24] recently conducted a meta-analysis on the effects of TC/Yoga on HRV parameters. Researchers concluded that subgroup analysis for TC was not recommended because only three randomized controlled trials (RCT) investigated the beneficial effects of TC for HRV, particularly with different parameters [24]. Due to the paucity of existing data, the current study explored the effects of TC on depression and time-domain of the HRV.

## 2. Methods

### 2.1. Study Participantsand Randomization

**Figure 1:**
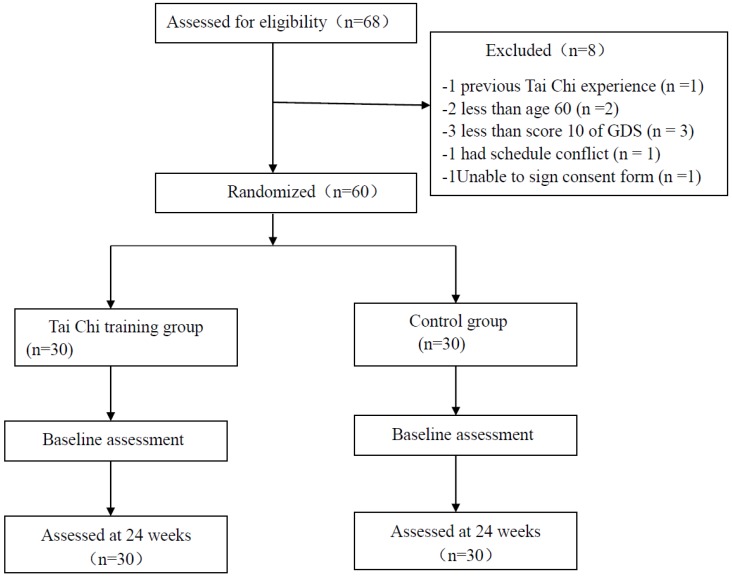
Flow diagram of eligibility assessment, exclusion, inclusion, and analysis.

Older adults who were not receiving antidepressant drugs and did not consume alcohol were recruited from several senior citizen centers in Shanghai, China. Only participants who met the following criteria were considered eligible: (1) age 60 or above; (2) scored 10 or above according to the Geriatric Depression Scale (GDS) administered by a certified psychologist; (3) did not participate in any structured exercise program in the past six months. Participants were excluded if they: (1) had any major disease such as mental disorders other than depression, heart disease, diabetes, hypertension, alcohol/drug addiction, comorbid psychotic disorder, and/or kidney disease; (2) were physically unable to perform TC movements; and (3) had no previous TC experience. According to the mention-above eligibility criteria, sixty participants were included in this study and then randomized (a random number generator) into either a Tai Chi group (*n* = 30, female = 16, 60.9 ± 4.28 year) or control group (*n* = 30, female = 16, 61.72 ± 3.54 years). Table 1 shows the demographic information of the study participants. The flowchart presents the process of participant selection and experimental implementation (Figure 1).

### 2.2. Tai Chi Intervention Program

Prior to starting the experiment, the study was approved by the Institutional Review Board at Shanghai University of Sports and participants provided informed consent. Participants in the Tai Chi group exercised three times per week (60 min/session) for 24 weeks. Participants in the control group were asked to maintain their unaltered lifestyle without engaging in any structured exercise program.

In the experimental group, participants received both 24- and 42-style TC forms (each site taught both 24 and 42 style). The number of movements in 24-style Tai Chi form need to be mastered prior to learning 42-style Tai Chi routine. In other words, although both Tai Chi styles have shared the similar movements, 24-style Tai Chi is a foundation of 42-style Tai Chi. It tells us that training regime administered by Tai Chi instructors should be from easy (24-style) to difficult (42-style). As mentioned previously, participants in the TC program received three 60-min sessions weekly for 24 weeks (October 2009 to April 2010). Classes were taught by a TC master with more than 10 years of teaching experience. The TC intervention involved three phases in which the specific information participants were instructed to concentrate. In phase 1 (4 weeks), participants were instructed to familiarize themselves with TC movements while breathing technique and relaxation skills were taught along with TC music. In phase 2 (12 weeks), the instructor emphasized the quality of physical components through assessing movement fluency and whole-body coordination. In phase 3 (8 weeks), participants were instructed to perform flowing and relaxed movement coordinated with deep breathing and mental concentration. Each instructor-led TC session involved a 10-min warm-up (muscular stretching for injury prevention), a 40-min TC form training, and a 10-min cool-down (flapping and bending bodies, deep breathing, meditation), and it occurred between 6:30 am to 7:30 am at the senior citizen center. 

### 2.3. Outcome Measures

In this study, we measured both behavioral outcome and HRV parameters. Depressive symptoms were measured using the GDS. The HRV measure is considered non-invasive. Given that little research has investigated the effects of mind-body exercises (TC, Yoga, and Qigong) on frequency domain of HRV, to fill this gap, we included both frequency- and time-domains. The baseline and post-intervention assessments were administered by experts (physicians for HRV) who were blinded to group assignment.

#### 2.3.1. Depressive Symptom Measured by the Geriatric Depression Scale

Depressive symptom was measured using the Geriatric Depression Scale (GDS) [25,26]. The self-reported GDS consisted of 30-item questionnaire, which has been tested and used extensively with the older individuals. Participants were asked to respond by answering “yes” or “no” regarding their perception in the last week. Of 30 items, 20 of them were positive responses (yes = 1 point), whereas the remaining 10 items were negatively scored answers (No = 1 point rather than “Yes”). The questionnaire usually demands 10 min to complete, with higher scores indicating worse depression (10 points or below = normal; between 10 and 19 = mild depression; 20 points or above = severely depressed).

#### 2.3.2. Both Time- and Frequency-Domains of Heart Rate Variability

All participants were informed that caffeinated beverages and tobacco smoking were prohibited for 12 h before HRV measurement in the morning. Participants were asked to quietly sit upright with normal breathing and rest for 20 min before the electrocardiogram (ECG) recording started for 5 min at a sampling rate of 1 kHz (PowerLab and Chart software, ADI Instruments, Castle Hill, Australia). Power spectral analysis involved fast Fourier transform [27]. Time-domain HRV metrics were computed according to 300 R-R intervals of the task force standard, including average heart rate (normal-to-normal intervals in beats/min, the standard deviation of normal-to-normal R-R interval (SDNN, overall HRV in millisecond), the root mean square of the beat-to-beat differences (RMSSD in millisecond, average magnitude of heart rate changes between consecutive beats indicates a marker of vagal heart rate modulation), and percentage of successive RR intervals that differ by more than 50 milliseconds (pNN50). For the HRV analysis in the frequency domain, 5-min long R-R time series were interpolated at 250 milliseconds to obtain equidistant values. Frequency bands in normal units (ms^2^) were obtained, including very low frequency (VLF) power (0.003 to 0.04 Hz), low frequency (LF) power (0.04 to 0.15 Hz), and high frequency power (0.15–0.4 Hz), and total power. In addition, LF/HF ratio (%) was individually computed, whereas LF and HF were normalized. 

### 2.4. Statistical Analyses

Firstly, categorical data and continuous data of demographic information were analyzed using Chi-square tests and independent *t*-test, respectively. Secondly, paired *t*-tests were conducted to determine any difference in all variables of interest between baseline and post-intervention tests. Thirdly, mean change for each outcome variable was obtained by subtracting the value measured at baseline from the measurements taken at Week 24. And then we performed *t*-tests to determine group differences in the mean change of depression and HRV parameters. To investigate the relationship between depression and HRV parameters, multiple Pearson Product-Moment Correlation Coefficient (PPMCC) were performed based on mean change scores of each outcome. Statistical significance was accepted at the threshold of *p* < 0.05. All values are expressed as means and standard deviations.

## 3. Results

A total of 68 older individuals were initially recruited and then they were screened against the eligibility criteria. In the screening six participants were excluded because they did not meet the inclusion criteria, one participant could not participate due to schedule conflict, and one participant was unwilling to sign the consent form. Thus, sixty participants were finally included in this study. No significant difference for demographic information was observed between the TC and control group (Table 1).

Statistically significant group differences were observed for change over time for depression, M-HRT, RMSSD, HF, LFnorm, and HFnorm (*p* < 0.05). Furthermore, when TC group demonstrated significant reductions in depression, M-HRT, and LFnorm (*p* < 0.05), no significant within-group difference on these outcomes were observed in the control group. In addition, when TC group demonstrated significant increases in RMSSD, HF, and HFnorm (*p* < 0.05), no significant within-group differences on these outcomes were observed in the control group (Table 2).

Depression measured by the GDS was significantly negatively associated with HF power (*p* < 0.01), whereas this behavioral outcome was significantly positively associated with very low frequency power (*p* < 0.05). Correlation results are presented in both Table 3.

## 4. Discussion

The results of the present study indicated that 24-weekTC program (60 min/sessions, three sessions per week) was effective in reducing depression among the elderly. Furthermore, researchers investigated the potential neurophysiological mechanism of alleviative effects of TC through analyzing HRV parameters. Positive results indicated that TC may have the potential to effectively modulate these HRV parameters, which possibly contributes to the equilibrium of the autonomic nervous system for alleviating depression. The results suggest that TC could be an appropriate exercise for the community dwelled elderly to alleviate their depression. More details about these positive results will be discussed below.

Geriatric depression is a mental and emotional disorder affecting older individuals [28]. Specifically, age-related complications (limited mobility, isolation, facing mortality, transitioning from work to retirement, and deaths of family members) lead to a strong possibility of developing depression in the elderly [29,30,31]. Thus, it is very important to offer some late-life leisure activities for the elderly to strengthen their physical functioning, reduce loneliness sand/or fear of death, and maintain social connections with other individuals. Tai Chi is a leisure activity that is suitable for the older individuals with low exercise tolerance [32]. In addition, Tai Chi is a mild-to-moderate exercise that helps elderly individuals attenuate the progressive decline of age-related cognitive and physical functioning [33,34], and it provides a platform where these older adults can communicate with each other both inside and outside of TC classroom, reducing social isolation [35]. Thus, the significantly reduced depression following the 24-week TC training seems to be reasonable. Positive results of this study are also supported by other studies [36,37,38,39,40,41] that investigated the alleviative effects of TC or Qigong exercise for depressive symptoms. For example, a randomized controlled trial by Chan et al. [41] investigated the effects of Baduanjin Qigong exercise on depression in women with chronic fatigue syndrome-like illnesses. The results of this well-designed study indicated that decrease in depressive levels were significantly associated with increases in adiponectin level after 1690 min Qigong sessions. The researchers concluded that the Qigong may produce anti-depressive effects on depression through upgrading adiponectin levels [41].

In addition to this behavioral measure, we also investigated the effects of TC on parameters, because HRV is known to decrease as people age [42]. A review by Rotenberg indicated that 312 people with depression showed significantly lower HRV than a matched control group of 374 healthy people [43]. In the present study, the 24-week TC program resulted in higher RMSSD, HF, and HFnorm, but lower mean heart rate and LFnorm. The RMSSD is the primary time-domain measure used to estimate the vagally meditated changes [44] and it is strongly associated with the HF power/HFnorm [45]. It is well-documented that change in HF component of HRV depends on respiratory rhythm [46]. It is worth emphasizing that one of the key components in TC is breathing technique. The 24-week TC may provide the opportunity with older individuals with depression to chronically adapt breathing patterns and regulate parasympathetic activity, which may elevate both RMSSD and HF/HFnorm. Such results are in great demand in the current literature because a meta-analysis paper by Zou et al. [24] indicated that only four of the 11 studies (three studies with Yoga and one study with Tai-Chi) reported the significant benefits on the HFnorm. In addition, reduced mean heart rate wasfound in this study. TC involved muscular relaxation with mental concentration, which may effectively regulate mental state [47]. This modulation possibly contributes to balance between sympathetic and parasympathetic systems, reducing heart rate. Positive results of this study were further confirmed by correlation tests. For example, depression in the TC group was negatively related to HF power, but positive relations with very low frequencies.

We would like to acknowledge some limitations in this study. Both participants and the instructor were not blinded. Thus, participants may have come to TC classes with greater expectations and instructors were paid much more attention when they were aware of the importance of this TC intervention. Second, the relatively small sample size cannot rule out the possibility that the positive findings emerged by chance, instead of the TC intervention. Third, this study lacked attention control that is usually employed in behavioral intervention research in order to control for nonspecific effects (e.g., attention, treatment contact, social support, and nonspecific instructor effects) [48]. Fourth, the follow-up assessment was absent, so we could not determine how long the positive effects of TC for reducing depression last. 

## 5. Conclusions

The results of this study indicated that TC may alleviate depression in the elderly through modulating autonomous nervous system or HRV parameters. This study added to a growing body of research showing that TC may be effective in treating depression of the elderly. Tai Chi as a mild to moderate mind-body exercise is suitable for older individuals who suffer from depression.

## Figures and Tables

**Table 1:** Demographic characteristics of all subjects in both the Tai Chi and control groups.

Variable	Tai Chi (*n* = 30)	Control (*n* = 30)	*p*
Female (%)	53.3	53.3	1.00 *
Age (year)	60.90 ± 4.28	61.72 ± 3.54	0.37 ^#^
Body weight	60.73 ± 2.14	57.59 ± 1.45	0.28 ^#^
Height	164.33 ± 2.09	165.28 ± 0.95	0.52 ^#^

Note: * Chi-square test; ^#^ Independent *t*-test.

**Table 2:** Effects of Tai Chi on depression and heart rate variability.

Parameters	Tai Chi		Control	
	Pre (*t* = 0)	Post (*t* = 24 Weeks)	Pre (*t* = 0)	Post (*t* = 24 Weeks)
GDS	11.97 ± 4.32	4.70 ± 3.90 *	12.00 ± 3.08	12.40 ± 3.38 ^▲^
Time Domains				
M-HRT (bpm)	82.26 ± 9.85	74.81 ± 6.58 *	81.85 ± 9.75	80.69 ± 7.57 ^▲^
SDNN (ms)	32.33 ± 15.21	37.24 ± 1.02	33.95 ± 12.27	31.73 ± 1.66
RMSSD (ms)	10.27 ± 6.67	15.97 ± 5.35 *	10.69 ± 5.97	10.05 ± 7.07 ^▲^
pNN50 (%)	0.64 ± 1.55	1.04 ± 1.15	0.67 ± 1.36	0.54 ± 1.06
Frequency Domains				
TP (ms²)	149.22 ± 156.36	167.33 ± 48.71	150.76 ± 113.32	146.22 ± 152.40
VLF (ms²)	53.96 ± 43.13	49.11 ± 11.18	50.51 ± 33.56	55.56 ± 13.82
LF (ms²)	80.41 ± 25.52	70.48 ± 12.45 *	77.57 ± 17.26	81.14 ± 42.39
HF (ms²)	22.21 ± 17.50	34.30 ± 12.45 *	24.07 ± 8.19	21.92 ± 23.72 ^▲^
LF/HF	2.32 ± 1.21	1.90 ± 0.99	2.41 ± 1.01	2.58 ± 1.10
LFnorm	82.00 ± 9.38	75.48 ± 9.74 *	79.71 ± 10.72	81.08 ± 11.20 ^▲^
HFnorm	19.56 ± 7.92	25.01 ± 6.51 *	20.62 ± 8.93	18.91 ± 11.20 ^▲^

Note: M-HRT = mean heart rate; SDNN = the Standard deviation of normal-to-normal R-R interval; RMSSD = the root mean square of the beat-to-beat differences; PNN50 (%) = Percentage of successive RR intervals that differ by more than 50 millisecond; TP = total power; VLF = very low frequency power; LF = low frequency power; HF = high frequency power; LF/HF = low frequency power to high frequency power ratio; LFnorm = low frequency in normalized unit; HFnorm = high frequency in normalized unit; Values are expressed as mean ± SD. Difference between baseline and week 24 for Tai Chi Chuan and control groups: * *p* < 0.05; group difference in mean change (from Week 24 to baseline): ▲ *p* < 0.05.

**Table 3:** Correlation analysis between depression and HRV parameters in mean change.

HRV Measures	M-HRT (bpm)	SDNN (ms)	RMSSD (ms)	pNN50 (%)	TP (ms^2^)	VLF (ms^2^)	LF (ms^2^)	HF (ms^2^)	LF/HF	LFnorm (nu)	HFnorm (nu)
*r*	0.16	−0.06	−0.17	−0.03	0.01	0.30	0.14	−0.38	0.10	0.23	−0.15
*p*	0.21	0.64	0.2	0.82	0.97	0.02 *	0.28	0.00 **	0.44	0.08	0.27

Note: M-HRT = mean heart rate; SDNN = the Standard deviation of normal-to-normal R-R interval; RMSSD = the root mean square of the beat-to-beat differences; pNN50 (%) = Percentage of successive RR intervals that differ by more than 50 millisecond; TP = total power; VLF = very low frequency power; LF = low frequency power; HF = high frequency power; LF/HF = low frequency power to high frequency power ratio; LFnorm = low frequency in normalized unit; HFnorm = high frequency in normalized unit. * *p* < 0.05, ** *p* < 0.01.
